# Focus characterization of the NanoMAX Kirkpatrick–Baez mirror system

**DOI:** 10.1107/S1600577519003886

**Published:** 2019-06-03

**Authors:** Markus Osterhoff, Anna-Lena Robisch, Jakob Soltau, Marina Eckermann, Sebastian Kalbfleisch, Dina Carbone, Ulf Johansson, Tim Salditt

**Affiliations:** a Institut für Röntgenphysik, Universität Göttingen, Friedrich-Hund-Platz 1, 37077 Göttingen, Germany; b MAX IV Laboratory, Lund University, Fotongatan 2, 22484 Lund, Sweden

**Keywords:** nano-focus, coherence, holography

## Abstract

The focusing and coherence properties of the NanoMAX Kirkpatrick–Baez mirror system at the fourth-generation MAX IV synchrotron in Lund have been characterized. The direct measurement of nano-focused X-ray beams is possible by scanning of an X-ray waveguide, serving basically as an ultra-thin slit. In quasi-coherent operation, beam sizes of down to 56 nm (FWHM, horizontal direction) can be achieved. Comparing measured Airy-like fringe patterns with simulations, the degree of coherence |μ| has been quantified as a function of the secondary source aperture (SSA); the coherence is larger than 50% for SSA sizes below 11 µm at hard X-ray energies of 14 keV. For an SSA size of 5 µm, the degree of coherence has been determined to be 87%.

## Abstract 2

Using an X-ray waveguide, the focal spot size and coherence properties of the NanoMAX Kirkpatrick–Baez mirror system have been measured.

## 1. Introduction   

X-ray beams are a ubiquitous tool in many areas from biological physics and chemistry to material science and the semiconductor industry. Broad experimental techniques range from absorption spectroscopy and fluorescence mapping, crystallography and scattering to imaging. While the spatial resolution lies between that of optical and electron microscopy, X-rays feature a large penetration length in matter. Therefore, imaging can be combined with computed tomography, thus providing three-dimensional views of small samples revealing features of less than 1 µm in biological specimens, and less than 100 nm in non-organic matter (Cloetens *et al.*, 1995 ▸, 1999 ▸; Jiang *et al.*, 2010 ▸; Guizar-Sicairos *et al.*, 2011 ▸; Krenkel *et al.*, 2013 ▸; Töpperwien *et al.*, 2018 ▸; Müller *et al.*, 2018 ▸).

Dedicated synchrotron experiments provide well-controllable beams for certain applications, often with tunable wavelength/photon energy. At the NanoMAX beamline of the MAX IV synchrotron, for example, diffraction, scattering and fluorescence are combined as imaging techniques to answer questions in material science, life and earth science, general physics, but also chemistry and biology (Johansson *et al.*, 2013 ▸). The beamline consists of two experimental hutches. The first will use Fresnel zone plates for focusing, but is still under development. In the second experimental hutch, the X-ray beam is focused by mirrors in Kirkpatrick–Baez (KB) geometry (Kirkpatrick & Baez, 1948 ▸) to beam sizes of 70 nm and smaller.

Here we present a characterization of focal spot size and coherence properties of the horizontally focused beam. The spot size and its dependence on (i) KB mirror tilt angle, (ii) coherence properties defined by a pair of slits defining the secondary source size, and (iii) X-ray photon energy are quantified. The degree of coherence, defined by the visibility of fringes, has been measured as a function of secondary source size.

The paper is organized as follows. First, we briefly describe the beamline and essential components, especially the optical system including KB mirrors and secondary source aperture. In Section 2, the applied experimental method is described and compared with other techniques; then the beam size and coherence properties are reported. In Section 3, the focal spot size and lateral shift are quantified for different X-ray photon energies. The paper closes with a summary and outlook.

### 1.1. MAX IV and NanoMAX beamline layout   

The NanoMAX beamline (Vogt *et al.*, 2017 ▸) at MAX IV is an undulator beamline at a 3 GeV synchrotron. The in-vacuum undulator with a magnet period of 18 mm can be closed to a gap of 4.2 mm, to access an energy range of 5–30 keV. The energy is selected by a horizontal double-crystal monochromator (HDCM, Si 111). Two mirrors focus the undulator beam into a secondary source aperture (SSA) 51 m downstream from the undulator source and 47 m from the nano-focusing endstation.

During the experiment, the MAX IV synchrotron was operated at the nominal electron energy of 3 GeV, with an electron beam current between 130 and 170 mA, with a life time of about 23 h. The top-up mode was set to about 1% and re-filled electrons with a period of 30 min. The primary X-ray energy of the beamtime was 14 keV and the undulator gap was closed to 5.21 mm, using its seventh harmonic. The experimenters were able to quickly change to 10 keV and 18 keV using the monochromator only, and ‘jumping’ between undulator harmonics.

**Figure 1:**
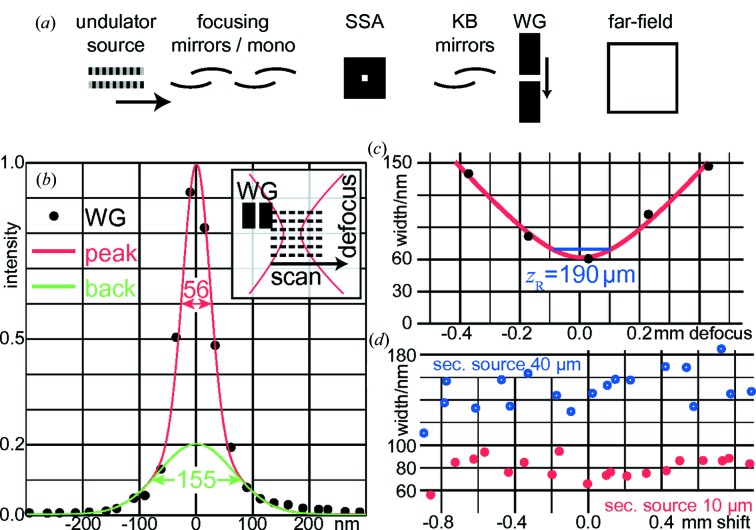
(*a*) Simplified layout of the NanoMAX, showing undulator, focusing mirrors, secondary source aperture (SSA), focusing mirrors (KB), the waveguide (WG) as a direct intensity probe in the focal plane, and the far-field detector. (*b*) 1DWG scan of the X-ray focus, fitted with two Gaussians with a 56 nm (FWHM) peak on a 155 nm background, for a horizontal SSA size of 10 µm. (*c*) Gaussian waist fits of a defocus series of 1DWG scans for one mirror pitch setting. (*d*) Minimum beam waists obtained from (*b*) for different mirror pitch angles, shown as a function of the corresponding longitudinal focal plane shift. Lower (red) values correspond to an SSA size (horizontal) of 10 µm, higher (blue) values show data for an SSA size of 40 µm.

For X-ray detection, both a single-photon-counting Pilatus 100k detector (Dectris Inc., Baden, Switzerland), placed about 3.73 m downstream of the focus, and an imaging sCMOS camera with Gadox scintillator (Photonic Science Ltd, Robertsbridge, UK) at about 2.52 m were employed. The instrumentation both for the waveguide and the sample was based on the Göttingen multilayer zone plate scanning setup (Osterhoff *et al.*, 2017 ▸). A simplified layout of the beamline including the waveguide is shown in Fig. 1 ▸(*a*).

### 1.2. Kirkpatrick–Baez mirror system   

The KB mirrors are situated in the second experimental hutch, at a distance of 98 m downstream from the undulator, or 47 m downstream from the SSA. The mirrors have an active length of 140 mm (vertically focusing mirror, VFM) and 90 mm (horizontally focusing mirror, HFM), and operate at an incident angle of 2.7 mrad (VFM) and 2.5 mrad (HFM); the focal lengths are 310 mm (VFM) and 180 mm (HFM). The actual focal planes can be shifted individually by changing the mirrors’ pitch angle; to avoid astigmatism, both planes need to coincide. The KB mirrors were fabricated by JTEC Corporation (Osaka, Japan) with a figure error of less than 1 nm and a Pt coating for high reflectivity up to a photon energy of 25 keV. The divergence angle of the focused beam is about 1.22 mrad × 1.25 mrad.

### 1.3. Secondary source aperture   

Coherence properties are primarily determined by the source emittance, but can be adjusted with slits.

The MAX IV synchrotron is a fourth-generation source, which for the first time implements a multi-bend achromat (MBA) as magnetic lattice (Tavares *et al.*, 2014 ▸, 2018 ▸). This allows for a reduced horizontal emittance of about 330 pm rad and a vertical emittance of better than 8 pm rad. The undulator source has a finite size of about 100 µm × 5 µm [full width at half-maximum (FWHM), H×V]. Although the horizontal emittance is about one order of magnitude smaller than for third-generation sources, the nano-focusing optics in the experimental hutch would still not be illuminated by a perfect coherent wave. To adapt the coherence length, an SSA is installed 51 m behind the undulator. The undulator beam is focused into the SSA by a sagittally focusing mirror with a fixed-curvature circular cylinder and a meridionally focusing bendable mirror. Both mirrors deflect the beam horizontally.

The horizontal and vertical opening gaps of the SSA can be controlled independently. This allows us to vary the coherence length, in order to match the geometrical acceptance of the KB nano-focusing optics at the experiment, and achieve spot sizes limited by diffraction only. On the other hand, by opening the slits, a larger spot size with highly increased flux can be obtained.

With the SSA, the coherence properties can be manipulated, without affecting the numerical aperture of the KB mirror system. The coherence length ξ scales linearly with wavelength λ and inversely with the SSA size *s*, *i.e.* 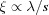. See Table 1 ▸ for a summary of the terminology used throughout this paper (*i.e.* ‘quasi-coherent’ versus ‘partially coherent’) and the respective SSA sizes in the horizontal direction.

### 1.4. X-ray waveguides   

X-rays, like all electromagnetic waves, can be geometrically confined by a lateral index of refraction profile *n*(*x*) with *n*_inside_ > *n*_outside_. For X-rays, this can be achieved by a channel/layer of low-*Z* material (or air, vacuum) placed inside a high-*Z* cladding material. Geometrically, the beam is kept inside the low-*Z* region by total reflection. In the wave picture, the intensity is confined as a guided mode inside a trough given by the electron density; only a discrete set of modes exists (Pfeiffer *et al.*, 2002 ▸; Bergemann *et al.*, 2003 ▸; Osterhoff & Salditt, 2009 ▸).

Here, the waveguide (WG) structure is made from a very thin guiding layer of 35 nm Co sandwiched between Mo layers of 30 nm thickness on a Ge substrate. Two such one-dimensional channels are mounted perpendicularly, such that both horizontally and vertically confined channels can be accessed individually, and an effective two-dimensional WG is realized at their crossing. The one-dimensional structures have lengths of 220 µm and 270 µm (Salditt *et al.*, 2008 ▸).

The WG channels have to be aligned with respect to the X-ray beam such that a maximum throughput of photons is achieved. To this end, the horizontally and vertically confined WG channels can be independently rotated in a tip/tilt direction. The horizontally confined channel, for example, has to be rotated along the vertical direction in order to achieve maximum throughput of photons; the angular acceptance is given by the critical angle of total reflection – here 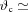 1.01 mrad and is thus compatible with the mirrors’ numerical aperture. The accuracy for alignment needs to be better than 50 µrad. With the horizontally confined channel, the horizontal focus profile can be measured by horizontal scans [see the inset in Fig. 1 ▸(*b*)].

## 2. Focus characterization   

One-dimensional X-ray waveguides (1DWG) have been used as ultra-thin slits and scanned through the X-ray beam in the focal plane and at several defocus positions along the beam waist. The principle is shown schematically in the inset of Fig. 1 ▸(*b*). To first approximation, only the guiding layer of 35 nm thickness collects X-rays, and hence the transmitted intensity quantifies the local intensity in the X-ray spot. From the one-dimensional step-scan and the total intensity transmitted to the Pilatus 100k detector, the intensity distribution of the KB system in the focus and defocus can be accessed directly.

Apart from WG scanning, a lot of other techniques are known. In the following, we will briefly address these techniques before reporting on the results achieved by WG-aided focus and coherence characterization.

### 2.1. Experimental determination of focus sizes   

*Ptychography* is a well-established technique to reconstruct the complex-valued sample transmission function as well as the illuminating field. It is based on a raster-scan with large overlaps and numerical phase retrieval supported by these overlaps (Faulkner & Rodenburg, 2004 ▸; Thibault *et al.*, 2008 ▸; Maiden & Rodenburg, 2009 ▸; Kewish *et al.*, 2010 ▸). For ptychography to work, sampling criteria by the detector and the scan have to be met; also, a significant degree of coherence is needed. The latter limitation has been relaxed by the development of multi-mode reconstructions (Thibault & Menzel, 2013 ▸). Compared with our method, experimental setup and data analysis are much more involved.

*Speckle patterns* formed by the diffraction from colloids can be used to estimate beam size and coherence properties. The angular size of a speckle is given by λ/Δ, where λ is the wavelength and Δ the beam size in the focus. The intensity histogram of the speckle pattern can be described by an Erlang distribution, and the number of modes can be fitted as a parameter to this family of distributions (Gutt *et al.*, 2012 ▸; Mai *et al.*, 2013 ▸). For this method to work reliably, the colloids have to be illuminated by a sufficiently flat wavefront; hence, the sample has to be placed within the Rayleigh length of the focus and aberrations have to be minimized – but cannot be measured.

*Diffraction from a fibre* has been used by Kohn *et al.* (2000 ▸) to characterize the degree of coherence of an undulator beamline at the European Synchrotron Radiation Facility in Grenoble. X-rays are scattered off a fibre or a slit, and the visibility of fringes in the Fresnel region is evaluated. It is a very robust method carried out in the far-field of the source. It remains unable to determine the focal spot size.

*The Wigner function* emerges as a two-dimensional Fourier transform from the four-dimensional mutual intensity function (Mey *et al.*, 2014 ▸). It can be constructed from two-dimensional intensity measurements along the optical axis. All important beam parameters, including beam waist, Rayleigh length, divergence angles and beam quality factors 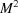 can be extracted from the four-dimensional function. The applicability to hard X-rays focused below 100 nm remains questionable, since the intensity distributions close to the focal plane are hard to measure.

*Hartmann wavefront sensors* consist of a plate of holes and a camera to record the diffraction pattern. They are typically used for soft X-rays, in a wavelength range from 10 to 40 nm, since for harder X-rays beam steering becomes negligible (Mey *et al.*, 2015 ▸).

*Waveguide scanning* has been successfully used to determine and tweak the hard X-ray focus at the GINIX instrument (Kalbfleisch *et al.*, 2010 ▸). It is a direct method to measure the intensity distribution in several planes along the optical axis. Because of the high signal-to-noise ratio, this method allows one to measure interference oscillations on the outer tails of the Airy-like fringe pattern; here we show that this modulation also holds information on the degree of coherence.

### 2.2. Horizontal intensity profile   

A typical WG scan is shown in Fig. 1 ▸(*b*). The black circles show the (normalized) transmitted intensity per scan point. To quantify the focal spot size, a sum of two Gaussians has been fitted. On top of a background Gaussian (FWHM of 155 nm, relative peak intensity of 0.2) lies a peak Gaussian with an FWHM of 56 nm.

For a more detailed analysis, the beam waist is modelled by a Gaussian beam. The WG scans are repeated over a total defocus region of ±1.2 mm in 13 planes. For the central planes, the evolution of the lateral beam size is obtained. With the usual definition of the Gaussian beam for the amplitude 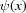,
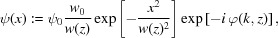
where 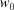 is the waist radius in the focal plane, the beam waist 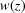 evolves as
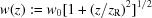
with Rayleigh length 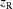, 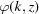 is the phase term and 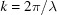 the wavenumber. Note that, at 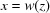, the amplitude drops to 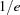. At 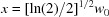, the intensity drops to 1/2, so FWHM_0_ and RMS_0_ (RMS = root mean square) of the intensity of the beam in the focal plane are given by
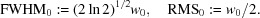
From the measured intensity profiles 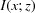 in different planes *z*, the 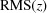 is calculated and converted to the Gaussian 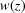. From fits, one obtains the best 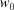 and 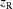 as well as the actual focal plane 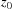 with the substitution 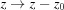. For a perfect Gaussian beam, however, 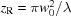. However, we do not impose this relation in the fits in order to cover deviations from the perfect beam, like finite coherence, aberrations and generally deviations from the Gaussian beam model.

The results are summarized in Fig. 1 ▸(*c*), showing the 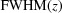 of five 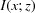 scans in different *z* planes and the Gaussian fit. From these fits, the best focal plane 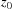 and respective beam size 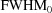 are obtained. The fitted Rayleigh lengths are on the order of 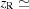 200 µm (SSA = 10 µm) and 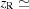 330 µm (SSA = 40 µm), and the WG scan planes are separated by 200 µm.

### 2.3. Best planes for varying mirror pitch angle   

The defocus series illustrated in the inset of Fig. 1 ▸(*b*) and with results given in Fig. 1 ▸(*c*) are repeated for different pitch angles of the HFM. From ray tracing, one can deduce that the change in pitch angle by 10 µrad yields a shift of the effective focal plane of the NanoMAX HFM system by about 720 µm. In Fig. 1 ▸(*d*), the spot sizes obtained from defocus scans such as the one in Fig. 1 ▸(*c*) are shown for a variety of HFM pitch values, plotted as a function of the focal plane shift along the optical axis. The red circles show data for the quasi-coherent case with the SSA closed to 10 µm, while the blue circles show data for the partially coherent case, with an SSA of 40 µm.

As can be seen, in the quasi-coherent case the focal plane can be shifted by more than ±0.8 mm with no significant effect on the spot size, which stays close to 80 nm. For the larger SSA, the spot size increases from about 80 nm to about 150 nm; again, for the shown HFM pitch variation of ±12 µrad, the achievable spot sizes remain within a small band.

### 2.4. Coherence properties   

**Figure 2:**
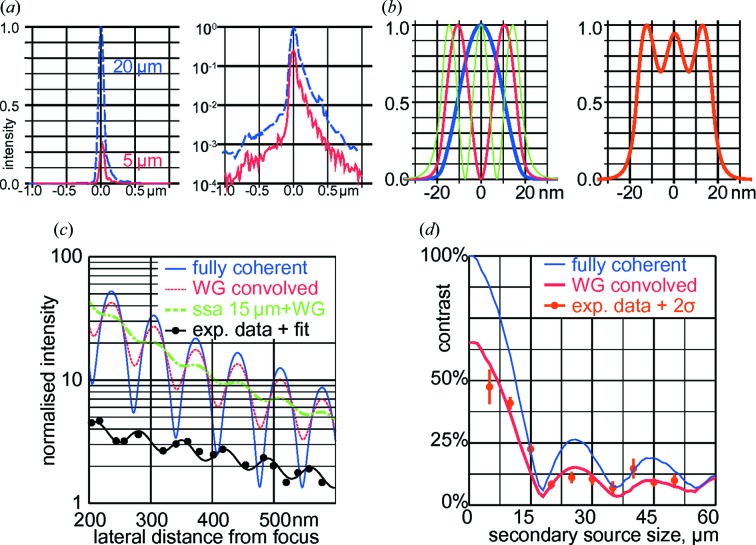
(*a*) Two measured focus profiles for a partially coherent setting (SSA size 20 µm, blue dashed curve) and a quasi-coherent setting (SSA size 5 µm, red line), shown on linear (left) and logarithmic (right) scale. (*b*) Intensity profiles of the three guided WG modes (left), superposition of the three modes (right). (*c*) Simulated and experimental focus tails, for different coherence settings. The blue line shows the simulated intensity of the HFM in a fully coherent setting; the red dashed line is convolved with the WG mode structure; the green line is calculated for a finite SSA size (horizontal) of 15 µm (and convolved with the WG guiding channel). The black circles represent experimental data for an SSA size of 15 µm, with a sinusoidal fit shown as a black line. Experimental data have been shifted by a factor of 1:3.5 vertically for clarity. (*d*) Fringe contrast, quantifying the degree of coherence, as a function of the SSA size. The thin blue line is simulated for ideal sampling, whilst the thick red line accounts for the convolution with the WG modes. The orange circles show contrast fits for experimental data; orange lines correspond to 2σ error bars of the fit.

Next, we address the coherence properties of the NanoMAX HFM focus. The degree of coherence can be traded for total flux by adjusting the SSA. It is defined via the contrast or visibility of interference fringes. With X-ray WGs, the near-field fringes can be measured at high dynamic range over more than three orders in magnitude. Here, we analyse the Airy-like fringes of the tails of the KB focus, at lateral distances of about 200 to 600 nm to the central peak. Fig. 2 ▸(*a*) shows two measured intensity profiles on a linear (left) and logarithmic (right) scale, for a partially coherent (blue dashed line) and quasi-coherent (red line) setting; the SSA sizes are 20 µm and 5 µm, correspondingly. The asymmetric profile, clearly visible on the log scale, is well known for KB beams with large numerical aperture: the varying angle of incidence along the mirrors yields a gradient in flux density and hence produces asymmetric beams.

When measured with a 1DWG of finite width, the actual intensity profile is convolved with the WG mode structure. Fig. 2 ▸(*b*) shows the three guided modes (left) and their sum (right). To quantify the degree of coherence, the experimental data have to be deconvolved with this mode structure. To accomplish this, the partially coherent intensity in the KB focus has been simulated. In Fig. 2 ▸(*c*), both simulated and experimental intensity data at these lateral distances are shown on a semi-logarithmic scale.

The simulations consist of two parts. First, the complex field in amplitude and phase for individual point sources placed along the SSA is calculated solving the Fresnel–Kirchhoff integral for 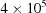 points along the mirror surface, including the respective Fresnel coefficients for reflectivity (Osterhoff & Morawe, 2010 ▸). Second, from the pre-calculated field amplitudes a partially coherent superposition is obtained by calculating an ensemble average of 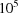 stochastic superpositions. The fields are weighted corresponding to the source size (Osterhoff & Salditt, 2012 ▸).

In Fig. 2 ▸(*c*), the blue line shows the simulated intensity profile assuming a perfect horizontally focusing mirror illuminated by a point source. The algebraic tail 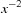 of the curve resembles the theoretical 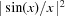 pattern of a one-dimensional focus. Note, however, that there are no true zeros in the intensity due to small aberrations induced by the elliptically curved mirror. In simulation, the intensity profile can be sampled with arbitrary precision; in the experiment, however, the intensity is convolved with the WG guiding layer of about 35 nm. Hence, the data from the mirror simulation are convolved with the expected mode structure of the WG, and shown as the red dotted line in Fig. 2 ▸(*c*).

For an aberration-free and fully coherent focus, the intensity profile is given as an Airy-like function of the form 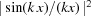. Note that for a ‘truly’ two-dimensional focus the numerator is given by a Bessel function, while the sine function holds for each one-dimensionally focusing mirror, which is addressed here with the WG scan. Now due to limited coherence, the interference fringes wash out. A fully analytical treatment, *e.g.* a convolution of the sine function by a Gaussian, tends to be rather cumbersome.

A more helpful approach has been chosen here, by using an empirical model function that resembles both the fast oscillating nature with variable amplitude due to limited coherence, and the long-range tails typical of KB mirrors. In addition, secondary effects such as surface scattering and *e.g.* air scattering need to be taken care of. Note that the oscillations are under-sampled, and hence from the data it is not possible to discern the ‘true’ convolved Airy-like function from a much more handy empirical fit function.

To quantify the contrast *c*, we use here the empirical model function,
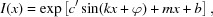
to fit the data. The absolute (or ‘raw’) contrast is fitted as 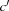; this is normalized to the relative contrast 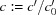, where 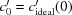 is obtained from simulated data. To this end, a point source has been propagated onto the KB mirror and the empirical function has been fitted for the same interval along the lateral axis.

The phase φ of the sine function is needed to define the origin of the axis. The additional term 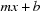 is used to include the expected 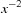 intensity tails, but also to capture additional background intensity.

The WG-convolved curve shows a reduced contrast by about 65%. This means that, even for a perfectly coherent source, the WG scan would show a reduced contrast; using these simulations, however, the contrast can be ‘deconvolved’ to estimate the true degree of coherence 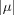 as a function of the SSA size *s*, here defined as
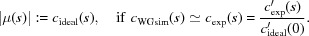
The NanoMAX beamline with its SSA allows the coherence properties to be changed independently from the numerical aperture of the mirror system. In our simulation, we also accommodate a finite SSA size, to steer the partial coherence properties. The thick green line in Fig. 2 ▸(*c*) shows the simulated intensity profile for a finite SSA size of 15 µm convolved with the WG channel. For this setting, the contrast is reduced to about 13% compared with the fully coherent case at perfect sampling, and to about 60% compared with the same finite SSA size at perfect sampling.

Now, the black circles in Fig. 2 ▸(*c*) show the experimentally measured intensity profile, vertically shifted by a factor of 1/3.5 for clarity; the black line shows the sinusoidal fit. The measured contrast is about 22 ± 2%, and a bit higher than theoretically expected. Fig. 2 ▸(*d*) shows the experimental contrast values as orange circles (including error bars from the non-linear fits) at different SSA sizes between 5 µm (quasi-coherent) and 50 µm (full flux). The thick red curve shows the corresponding WG-convolved simulated data. Apart from two outliers, experimental and simulated values agree within 2σ, and the experimental values reflect the oscillating behaviour of the contrast with minima at 18 µm and 36 µm.

The thin blue line corresponds to the partially coherent mirror simulations with arbitrary sampling, used to de-convolve the WG-blurred data. As can be seen, for the SSA sizes of 5 and 10 µm, the measured and simulated contrasts of about 56% (experimental: 47 ± 7%) and 37% (experimental: 31 ± 3%) are in good agreement. From the partially coherent mirror simulations (blue line) we estimate the ‘true’ contrast, and hence the degree of coherence, at these SSA size settings, to be about 88% and 60%, respectively.

The first minimum of the contrast function occurs at 18.2 µm; the degree of coherence is above 50% for SSA sizes up to 11 µm at hard X-ray photon energies of 14 keV.

### 2.5. Discussion   

A similar experiment has been discussed by Bakos & Kántor (1961 ▸) in the optical regime. They measured the far-field diffraction pattern of a fixed slit *d*, illuminated by an adjustable secondary source slit *s*. In their analytical treatment, they employ the Young–Rubinowicz theory (Rubinowicz, 1917 ▸; Miyamoto & Wolf, 1961 ▸) and extend it to the case of partially coherent illumination. In short, the edge diffraction at slit *d* is quantitatively described with the formalism of the Cornu spiral; the fringe contrast from partial coherence is readily included by the degree of coherence μ between the slit edges, obtained from the van Cittert–Zernike (van Cittert, 1934 ▸; Zernike, 1938 ▸) theorem: for a slit of size *s* and two pinholes separated by distance *d*, the degree of spatial coherence 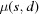 is given as
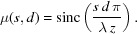
The intensity *I* in the geometrical shadow region of the slit *d* – here the aperture of the KB – is then given as
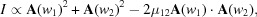
with
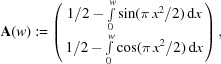
where 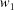 or 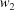 represents the path-length difference from the source via the slit edges 1 or 2 to the point of interest, compared with the direct beam (Bakos & Kántor, 1961 ▸).

To guarantee a robust fitting procedure, the simplified model function 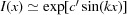 has been used to quantify the contrast 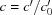. It can be shown that this fitting procedure successfully quantifies the degree of coherence for far-field diffraction curves obtained using the Bakos & Kántor method with parameters set to the HFM geometry considered here. To be more precise, the linear correlation coefficient of our estimated μ to the input degree of coherence is about 0.996, with an RMS error of about 1.3%.

With *d* ≃ sin(2.5 mrad) × 90 mm ≃ 225 µm as the projected geometrical aperture of the HFM, the first minimum of 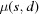 for λ = 8.86 × 10^−11^ m and *z* = 47 m occurs at *s* ≃ 18.5 µm, in good agreement with the experimental result of 18.2 µm.

The simple model by Bakos & Kántor does not predict the fringe contrast close to the minima of 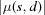 correctly. In fact, the contrast does not vanish completely, as has been shown *e.g.* by Shore *et al.* (1966 ▸) in experiments. Our simulations agree with their finding that the oscillations do not simply ‘flip over’, but are gradually shifting outwards while the focus size increases. The model gives, however, a comprehensible and short explanation.

## 3. Energy variation   

Certain experimental methods rely on – or can be improved by – a deliberate variation of the incidence X-ray photon energy. In general, this changes the index of refraction 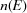. The effect can be highly non-linear and element specific close to absorption edges; details in 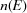 reveal chemical states in extended X-ray absorption fine structure (EXAFS). When used in a scanning setup with nano-focused beams, this chemical information can be spatially resolved as 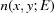. It is, however, necessary that the focal spot size and the spot position are not affected too strongly when the energy is changed, as this would reduce the resolution when 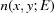 maps for different energies *E* cannot be directly compared.

In full-field holographic imaging, the anomalous scattering at different X-ray energies can be used to reformulate the interaction using the energy-independent electron density 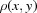, instead of the auxiliary quantity 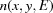. In addition, the effective Fresnel number changes. It is thus possible to collect data for *e.g.* holo-TIE [phase reconstruction using the transport of intensity equation (Cloetens *et al.*, 1999 ▸; Paganin, 2006 ▸) using a holographic defocus-series] (Krenkel *et al.*, 2013 ▸; Salditt *et al.*, 2015 ▸) without movement of the sample.

We have investigated the focal spot (i) size, (ii) position and (iii) intensity when changing the energy from 14 keV (seventh harmonic) to 18 keV (ninth harmonic), then again to 14 keV (seventh harmonic) and finally to 10 keV (fifth harmonic). To simulate an experiment under time constraints, the monochromator was just ordered to the nominal energy position; neither undulator nor monochromator have been tuned. After each energy change the beamline was given 2 min for stabilization. During that time the beam position was steered to its reference position as measured with a transmissive beam position monitor (NanoBPM, FMB Oxford) at the SSA. The closed-feedback loop acted on the fine-pitch and fine-roll angles of the second crystal of the HDCM. The PID feedback was set to very conservative values to reduce the risk of sudden beam jumps due to noisy readings of the beam position.

**Figure 3:**
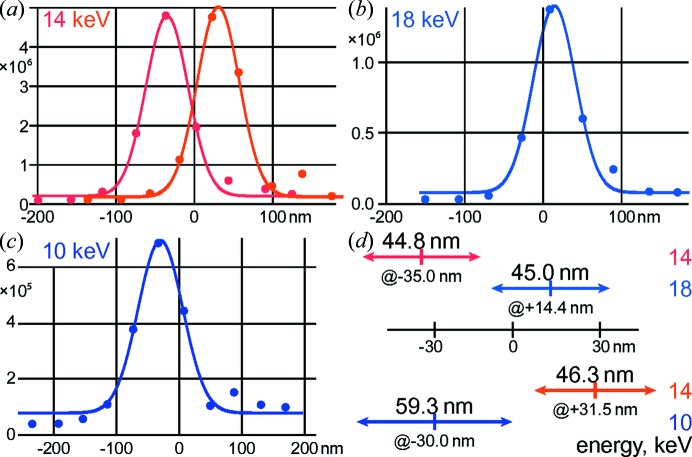
(*a*) 1DWG focus scan at 14 keV energy (repeated twice), (*b*) at 18 keV (without tuning), (*c*) at 10 keV (without tuning). (*d*) Comparison of beam size (FWHM from single-Gaussian fits) and peak position shift. The shifts can be partially attributed to drifts of the motorization stage.

At each energy, the intensity profile in the horizontal direction was WG-scanned in five longitudinal planes over ±200 µm. Figs. 3 ▸(*a*)–3 ▸(*c*) show the respective best scans for each energy. In Fig. 3 ▸(*d*), the relative widths (FWHM) and positions of the peaks are shown. Note that the repeated scans at 14 keV are separated by almost 70 nm; we attribute this to thermal drift of the WG stage, but cannot rule out residual beam movements induced by the monochromator crystals.

At the higher energy of 18 keV, see Fig. 3 ▸(*b*), the intensity is reduced by about a factor of three; this is expected due to the lower brilliance of the undulator at the higher harmonic. The focal spot size does not decrease, but is smeared out due to the reduced coherence length 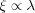. To fully utilize this energy in holography, the SSA needs to be closed accordingly.

For the lower energy of 10 keV, *cf*. Fig. 3 ▸(*c*), the intensity is much weaker. We attribute this to highly increased absorption both by the WG and by air; the latter could be normalized, of course. As a remedy, at lower X-ray energies, an evacuated beam pipe should be installed between the sample and detector. Compared with the scans at 14 keV, the spot size increases roughly linearly with wavelength.

## 4. Summary and outlook   

We have quantified the X-ray focal spot size and its dependence on (i) KB mirror tilt angle, (ii) coherence properties defined by the secondary source aperture, and (iii) X-ray photon energy for the new NanoMAX beamline at the MAX IV synchrotron. As a direct measurement technique, a 1DWG channel has been scanned directly through the X-ray beam, serving as an ultra-thin probing slit.

At 14 keV, a central peak size of 56 nm (FWHM) on top of a larger background has been obtained by a two-Gaussian fit. We have followed the beam waist for different mirror pitch angles and its longitudinal shift agrees with geometrical predictions. The focal plane can be shifted by at least ±0.8 mm with no significant effect on beam size.

The degree of lateral coherence |μ| of the focused beam is quantified from the fringe visibility in the Airy-like patterns of the focus tails; the experimental data agree very well with numerical simulations. These simulations also allow one to ‘deconvolve’ the finite-sized WG channel, and show a degree of coherence |μ| > 0.5 for secondary source aperture sizes below 11 µm.

## Figures and Tables

**Table 1:** Definition of terminology: degrees of coherence used in this paper and the respective secondary source aperture (SSA) sizes in the horizontal direction Limiting values 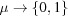 cannot be achieved experimentally.

Terminology	|μ|	SSA aperture (µm)
Coherent	1	0
Quasi-coherent	>0.5	<11
Partially coherent	<0.5	>11
Incoherent	0	
